# Deep Reinforcement Learning-Based Resource Allocation for Cellular Vehicular Network Mode 3 with Underlay Approach

**DOI:** 10.3390/s22051874

**Published:** 2022-02-27

**Authors:** Jinjuan Fu, Xizhong Qin, Yan Huang, Li Tang, Yan Liu

**Affiliations:** 1 College of Information Science and Engineering, Xinjiang University, Urumqi 830000, China; fujinj@stu.xju.edu.cn (J.F.); dandelion@stu.xju.edu.cn (Y.L.); 2 Network Department, China Mobile Communications Group Xinjiang Co., Ltd., Urumqi 830000, China; huangyan@xj.chinamobile.xh.com (Y.H.); tangli@xj.chinamobile.com (L.T.)

**Keywords:** vehicular network, deep reinforcement learning, resource management, low latency

## Abstract

Vehicle-to-vehicle (V2V) communication has attracted increasing attention since it can improve road safety and traffic efficiency. In the underlay approach of mode 3, the V2V links need to reuse the spectrum resources preoccupied with vehicle-to-infrastructure (V2I) links, which will interfere with the V2I links. Therefore, how to allocate wireless resources flexibly and improve the throughput of the V2I links while meeting the low latency requirements of the V2V links needs to be determined. This paper proposes a V2V resource allocation framework based on deep reinforcement learning. The base station (BS) uses a double deep Q network to allocate resources intelligently. In particular, to reduce the signaling overhead for the BS to acquire channel state information (CSI) in mode 3, the BS optimizes the resource allocation strategy based on partial CSI in the framework of this article. The simulation results indicate that the proposed scheme can meet the low latency requirements of V2V links while increasing the capacity of the V2I links compared with the other methods. In addition, the proposed partial CSI design has comparable performance to complete CSI.

## 1. Introduction

Vehicular communication is a vital realization technology of automatic driving and intelligent transportation systems [1]. Various candidate technical solutions have been proposed, such as cellular vehicular communication and dedicated short-range communication (DSRC) based on IEEE 802.11p [2,3]. DSRC has the disadvantages of short coverage distance and high infrastructure cost [4,5]. Despite the deployment of DSRC-based vehicular communication prototypes in the United States, inherent issues with DSRC and recent developments in cellular technology have encouraged research and industry research on cellular-based vehicular communications [6]. 5G Automotive Association (5GAA), as a global and cross-industry organization, was created in September 2016 and has presented the clear position of supporting cellular vehicular communication as the feasible solution for future mobility and transportation services [7]. Different regions such as the United States, Europe, and Japan have carried out pilot construction of cellular vehicular communication in actual operation [8]. Through cellular vehicular communication, road safety and traffic efficiency can be improved [9]. Therefore, cellular vehicular communication has attracted more attention from industry and academia. 3GPP Release 14 introduced modes 3 and 4 for vehicular communication [10]. These modes enable vehicles to directly communicate via licensed cellular frequency bands, bypassing the BS. Modes 3 and 4 both support direct vehicular communications, but their radio resource allocation methods are different. In mode 3, the BS allocates resources to vehicle users, while in mode 4, vehicles autonomously select radio resources [10,11]. Mode 3 can be divided into two types: overlay method and underlay method. In the overlay approach, V2I users and V2V users transmit in different frequency bands, while in the underlay method, V2V users reuse spectrum resources allocated to V2I users, which improves the spectrum utilization but interferes with V2I users. Therefore, in the underlay approach of mode 3 how to flexibly allocate resources to meet the low latency requirements of V2V users while increasing the throughput of V2I users is challenging.

## 2. Related Work

There have been more studies on the allocation of wireless resources for vehicular communication. The existing spectrum resource sharing schemes are mainly divided into two categories: traditional methods [12,13,14] and machine learning methods [15,16,17]. In conventional methods, under different constraints such as transmit power, quality of service, mathematical programming is used to optimize design goals of interest, such as maximizing spectrum efficiency or minimizing interference. Under normal circumstances, resource allocation problems are usually non-convex or mixed-integer nonlinear programming. Due to its NP-hardness, it is difficult to solve using effective methods. In order to deal with such problems, heuristic methods are usually used or sub-optimal solutions are pursued. In addition, the solution is highly dependent on the model. If you consider different objective functions or add new constraints, you need to develop a new solution [18]. Therefore, these many reasons have promoted the exploration of new resource allocation schemes.

In recent years, machine learning has become a potential implementation technology for future wireless communications. Reinforcement learning (RL) in machine learning is widely used in wireless communication resource allocation to solve the challenges in traditional optimization methods. In a high-speed moving vehicular network, RL can perceive changes through interaction with the environment and make corresponding decisions. In addition, the goal problems and constraints that are difficult to optimize can be solved by designing corresponding training rewards [19]. Q-learning is a typical RL algorithm, which avoids the difficulty of acquiring dynamics by adopting the iterative update method. However, since Q-learning stores the state in the form of a table, it is not suitable for application in scenarios with too large a state space. By combining deep neural network (DNN) with Q-learning, deep Q-learning (DQN) is designed to handle larger state spaces. In [20], the problem of vehicular network spectrum sharing based on multi-agent RL was studied, resource sharing was modeled as a multi-agent RL problem, and then the fingerprint-based DQN method was used to solve it. In [21], the spectrum allocation problem in the vehicular network was studied, and the graph neural network (GNN) and DQN learning were used to optimize the sum capacity of the vehicular network. In [22], the problem of shared resource allocation between V2I and V2V links is studied.

Under the deep reinforcement learning (DRL) framework, the existing resource allocation methods can be divided into modes 3 and 4. In the mode 3 scheme [23,24,25], the BS acts as a central controller to allocate resources for each user. In [23], a joint DRL algorithm was proposed, which can effectively deal with the discrete–continuous mixed action space in wireless communication systems and solve the power and sub-channel allocation problems of non-orthogonal multiple access systems. In [24], a resource allocation method based on graphs and DRL is proposed, in which channel resources are centrally allocated by BS, and the vehicle user uses DRL for distributed power control. In [25], the joint channel allocation and power allocation problem was transformed into an optimization problem, and a DRL framework based on an attention neural network was proposed to solve the problem. In the above-mentioned schemes, the BS needs to know the CSI of all users, that is, V2V user’s communication channel gain, the interference link information, and the user’s transmit power to determine the spectrum sharing decision. Feeding this information back to the BS will result in more significant overhead, especially when there are many users. This limits the application of the centralized solution in practice.

In the mode 4 resource scheme [26,27,28], the users independently select the transmission spectrum without the participation of the BS, which reduces the signaling overhead of the BS. In [26], a DRL-based V2V communication distributed resource allocation mechanism that can be used in both unicast and broadcast scenarios was developed to learn how to meet V2V latency constraints while minimizing the interference to V2I communication. In [27], a DRL-based mode selection and resource allocation method are proposed to maximize the total capacity of V2I users while ensuring the delay and reliability requirements of V2V users. In these schemes, each V2V pair independently updates its strategy at different times to maximize its reward. However, when two or more V2V pairs update their policies at the same time, it becomes a seemingly unstable environment. In [28], an effective transfer actor–critic learning approach was proposed to ensure the ultra-reliable and low-latency communication requirements of the V2V links, but this work can only be applied to low-dimensional state–action mapping. Using a distributed scheme can significantly reduce signaling overhead, but distributed DRL may lead to local optimization. Based on the research work analysis of the above-mentioned mode 3 and mode 4 resource allocation schemes, how to design an effective V2V resource allocation framework is the key to improving the data rate of V2I communication and meeting the requirements of V2V communication delay.

The analysis of the research work in [23,24,25,26,27,28] shows that DRL-based resource allocation with delay as the constraint is more common in the mode 4 scheme. However, due to the high signaling overhead, there are few studies on the mode 3 solution and primarily focus on solving the rate problem between V2I users and V2V users, without considering the delay requirements of V2V users. Compared with mode 4, the advantage of mode 3 is that the BS collects the CSI of all vehicles in the coverage area and can allocate spectrum resources more effectively. However, in a high-speed moving vehicle environment, it is a challenge for the BS to obtain complete CSI. Based on the above considerations, this paper proposes a DRL-based V2V resource allocation framework in the underlay of mode 3. In this framework, the BS uses double DQN for resource allocation. In particular, to reduce the overhead of CSI feedback, in the framework of this article, the V2V links only feedback partial CSI, and the BS only optimizes the resource allocation strategy based on the feedback information.

The main contributions of this work are summarized in the following aspects:

1. In view of the low latency requirements of V2V communication, a DRL-based resource allocation framework is proposed in the underlay method of mode 3, which aims to meet the V2V links latency requirements with low signaling overhead while increasing the V2I link throughput. It is novel to consider the delay requirement of V2V links in the DRL-based mode 3 spectrum allocation scheme.

2. To reduce the signaling overhead of BS acquiring CSI in the resource allocation scheme of mode 3, in this design, each V2V link only needs to feed back interference links information and delay information to the BS, instead of feeding back complete CSI signaling. The BS only optimizes the resource allocation strategy based on the feedback information, which significantly reduces the CSI feedback overhead.

3. The simulation results show that, compared with other solutions, the proposed solution can not only meet the low latency requirements of the V2V links but also increase the throughput of the V2I links, and the proposed partial CSI has comparable performance compared with the complete CSI.

The rest of the paper is organized as follows. The system model is introduced in Section 3. Section 4 presents the DRL framework to solve the problem formulation of spectrum resource allocation. The simulation results and analysis are shown in Section 5. Section 6 is the conclusion.

## 3. System Model

Since the uplink resources are under-utilized compared with the downlink ones [29], we only consider uplink cellular resource allocation.

**Figure 1:**
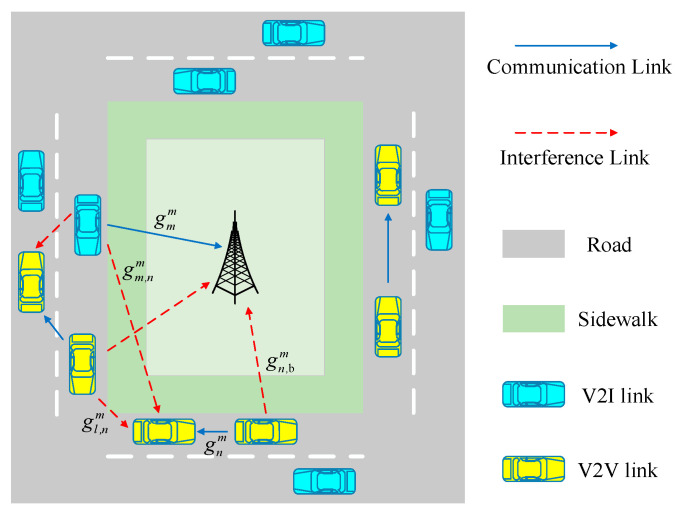
System model for vehicular communication underlaying cellular network in uplink.

As illustrated in Figure 1, an uplink scenario in a single cell system is considered. BS was lying in the center of the cell. The road configuration for vehicular communication is defined as an urban setting [30]. The cell consists of multiple V2I links, denoted as ***M*** = {1,…,M} and multiple V2V pairs denoted as ***N*** = {1,...,N}. In order to improve the spectrum efficiency and ensure the data rate requirements of the V2I links. We assume that each V2I user has assigned one orthogonal subcarrier resource and each V2I user allows more than one V2V pair to share its resource.

We define the channel power gain of the desired transmission for *m*th V2I link and *n*th V2V pair on the *m*th channel as $g_{m}^{m}$, $g_{n}^{m}$. Similarly, let $g_{m,n}^{m}$ represent the interfering channel power gain from the *m*th V2I transmitter to the V2V pair *n*’s receiver. The interference channel power gain between *n*th V2V link and the BS is denoted as $g_{n,b}^{m}$, and the interference channel gain from the V2Vpair *l*’s transmitter to the V2V pair *n*’s receiver is denoted as $g_{l,n}^{m}$. In the simulation in this article, all the channel gains mentioned above include path loss, shadow fading, and small-scale fading.

Then, we can respectively express the signal-to-interference-plus-noise-ratio (SINR) for the *m*th V2I link and *n*th V2V pair on subcarrier *m* as

(1) $$\gamma_{m}^{m}=\frac{p_{m}g_{m}^{m}}{\sum_{n=1}^{N}\rho_{n}^{m}p_{n}g_{n,b}^{m}+\sigma^{2}}$$

(2) $$\gamma_{n}^{m}=\frac{p_{n}g_{n}^{m}}{\sum_{l\neq n}^{N}\rho_{l}^{m}p_{l}g_{l,n}^{m}+p_{m}g_{m,n}^{m}+\sigma^{2}}$$

where *p_m_*, *p_n_*, *p_l_* denotes the transmit powers of the *m*th V2I, *n*th V2V pair, and *l*th V2Vpair. $\rho_{n}^{m}$ is the subcarrier access indicator, $\rho_{n}^{m}\in \left\{0,1\right\}$, $\rho_{n}^{m}=1$ if *n*th V2V pair is allowed to use subcarrier *m*, otherwise, $\rho_{n}^{m\;}=0$. *σ*^2^ indicates the noise power.

Furthermore, to reduce interference between V2V links, we assume each V2V link can only access one V2I link resource. Accordingly, the sum data rate of *m*th V2I link and *n*th V2V pair on subcarrier *m* can be respectively given by

(3) $$R_{m}^{m}=\log_{2}\left(1+\gamma_{m}^{m}\right)$$

(4) $$R_{n}^{m}=\log_{2}\left(1+\gamma_{n}^{m}\right)$$

We model the latency requirements of V2V links as the successful delivery of packets of size L within a limited time budget T_max_. Therefore, if the following constraint holds, the latency requirements of a V2V link will be satisfied:

(5) Pr{∑t=1Tmax∑m=1Mρn[m]Rnm[t]≥LΔT}

where ΔT is channel coherence time, and the index *t* is added in $R_{n}^{m}$[*t*] to indicate the capacity of the *n*th V2V link at different coherence time slots.

## 4. DRL for Resource Management

In this section, we describe how to use the DRL method within the proposed framework to solve the spectrum allocation, including the proposed partial CSI observations and double DQN learning algorithm.

For mode 3 resource allocation, our objective is to find the optimal resource allocation policy that can meet the latency demand of V2V links without excessively affecting the V2I links data rate under low signaling overhead. In addition, the vehicular communication network needs an intelligent resource management framework to make decisions intelligently, therefore, we adopt DRL architecture to make resource-sharing decisions. In the RL, an agent connects its environment, at each moment, the agent takes action based on the current environment state. The action will impact the state of the environment, causing certain changes in the environment. Then the agent receives numerical rewards and observes a new environment state. Through continuously exploiting the environment, agents learn how the environment generates rewards and then update their policy accordingly. In RL, the agent’s objective is to maximize the expected sum of rewards, which is defined as

(6) $$R_{t}=\sum_{k=0}^{\infty }\gamma^{k}r_{t+k+1}$$

where *γ* ∈ [0,1] is called the discounter factor.

**Figure 2:**
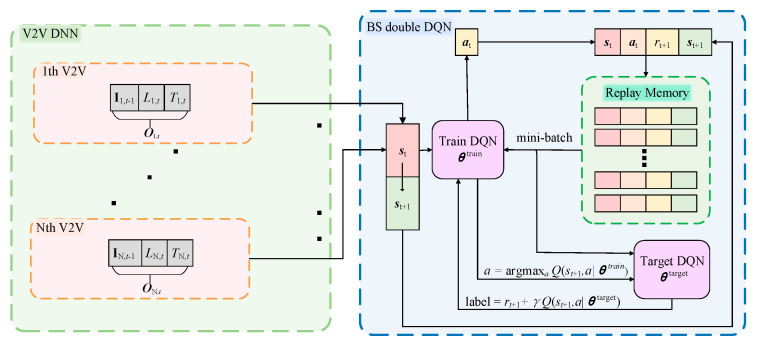
Proposed framework for resource allocation.

We adopt double DQN with experience replay architecture for subcarrier assignment, as shown in Figure 2. BS is regarded as a learning agent and the vehicular network acts as the environment state. Three key elements of the proposed model, i.e., state, action and reward, are defined, respectively, as follows.

As shown in Figure 2, each V2V pair observed local information, which includes communication interference channel power gain and latency information. We consider that it is difficult to obtain a complete CSI in the vehicle network, so only part of the CSI is considered in our observation space design. This helps to reduce the signaling overhead caused by channel estimation and CSI feedback. To consider the low latency communication requirements, key elements related to latency should be involved. In Figure 2, **I***_n_*_,*t*−1_, *L_n,t_*, *T_n_*_,*t*_ represents the interference power gain received by each sub-channel of the *n*th V2V link in the previous time slot, remaining payload size for transmission and the time left before violating the latency constraint; among them, $I_{n,t}^{m}$ can be written as follows

(7) $$I_{n,t}^{m}=\sum_{l\neq n}^{N}\rho_{l,t}^{m}p_{l}g_{l,n,t}^{m}+p_{m}g_{m,n,t}^{m}$$

In summary, the partial CSI observation space of the *n*th V2V pair can be expressed as

(8) $$o_{n,t}=\left\{I_{n,t-1},\;L_{n,t},\;T_{n,t}\right\}$$

where **I***_n_*_,*t*_=($I_{n,t}^{1}$, $I_{n,t}^{2}$, $I_{n,t}^{3}$,…, $I_{n,t}^{M}$).

Specifically, the complete CSI observation space is defined as

(9) $${\tilde{o}}_{n,t}=\left\{G_{n,t},\;I_{n,t-1},\;L_{n,t},\;T_{n,t}\right\}$$

where **G***_n_*_,*t*_=(**g***_n,t_*, **g***_m,n,t_*, **g***_l,n,t_*, **g***_n,b,t_*). Feeding back complete CSI to the BS will generate a large signaling overhead, and it is unrealistic in the dynamic environment of the vehicle, especially the links not connected to the BS, such as the V2V communication links and the interference of other vehicles on the V2V links. To obtain the **g***_n_*_,*t*_, **g***_m,n,t_* and **g***_l,n,t_*, channel estimation at the receiver side is necessary, after which the estimation results can be fed back to the transmitter. Then the transmitter feeds back to BS. Therefore, we consider not using complete CSI. On the other hand, in mode 4, the user selects the spectrum resource with sensing channel measurements, the level of interference that will be experienced if the sensing user transmits in the corresponding spectrum resource [31]. Therefore, we are motivated to consider designing a partial CSI resource allocation mechanism using interference power measurement. Compared with the complete CSI version designed as (9), the partial CSI observation space designed as (8) can help reduce the signaling overhead.

(1) State Space: As stated above, to reduce signaling overhead in a centralized solution, in our solution, each V2V link feeds interference and delay information back to the BS. Hence, BS considers the partial CSI feedback from all V2V pairs as the current state. Thus, the state space of the BS is described as follows

(10) $$s_{t}=\left\{o_{1,t},o_{2,t},o_{3,t},\ldots ,o_{N,t}\right\}$$

(2) Action Space: In this paper, we focus on the subcarrier assignment issues in the V2V communication network. Hence, the action is defined as

(11) $$a_{t}=\left\{b_{1,t},b_{2,t},b_{3,t},\ldots ,b_{n,t}\right\}$$

where *b_n_*_,*t*_ = *m*, ∀*m* ∈ M represents that the *m*th subcarrier has been selected by the *n*th V2V pair.

(3) Reward: In the RL framework, the training process is driven by the reward function. An agent searches decision-making policy by maximizing reward under the interaction with the environment. Hence, to meet the different requirements of communication devices, corresponding reward functions need to be formulated. In vehicular communications, V2V links exchange critical safety information and have strict latency requirements, whereas V2I links support high-rate data transmission [32]. Our objective is to improve the sum throughput of V2I links while meeting the latency requirements of V2V links. Therefore, we propose the following reward function

(12) $$r_{t}=\lambda_{c}\sum_{m=1}^{M}R_{m}^{m}\left[t\right]+\lambda_{v}\sum_{n=1}^{N}G_{n}\left[t\right]$$

where λ*_c_*, and λ*_v_* are the weight factors for the contributions of these two parts to the reward function composition. In response to the first objective, we simply include the sum capacity of all V2I links.

To achieve V2V link low latency requirements, for each V2V link, we set the reward *G_n_* equal to the effective V2V transmission rate until the V2V links deliver the payload successfully within the delay constraint, after which the reward is set to a constant number, *c*, that is greater than the largest possible V2V transmission rate. Therefore, the sooner the V2V link completes the transmission, the more rewards can be obtained. As such, the V2V-related reward at each time step *t* is

(13) $$G_{n}\left(t\right)=\left\{ \begin{array}{l} R_{n}^{m}\left(t\right)L_{n,t}>0\\ \;cL_{n,t}\leq 0 \end{array}\right.$$

Q-learning [33] is a typical RL algorithm, which can be used to learn the optimal strategy when the state–action space is small. In the Q-learning algorithm, the action-value function is used to calculate the expected accumulative rewards for starting from a state *s* by taking action *a* under policy *π*, which can be expressed by

(14) $$Q^{\pi }\left(s,a\right)=E\left[R_{t}|s_{t}=s,a_{t}=a\right]$$

Similarly, the optimal action-value function is obtained by

(15) $$Q^{*}\left(s,a\right)=E\left[r_{t+1}+\gamma \underset{a_{t+1}}{\max}Q^{*}\left(s_{t+1,}a_{t+1}\right)|s_{t}=s,a_{t}=a\right]$$

where *s_t_*_+1_ is the new state after taking action *a*. The action-value function is updated by

(16) $$Q\left(s_{t},a_{t}\right)\leftarrow Q\left(s_{t},a_{t}\right)+\alpha \left[r_{t+1}+\gamma \underset{a_{t+1}}{\max}Q\left(s_{t+1},a_{t+1}\right)-Q\left(s_{t},a_{t}\right)\right]$$

where *α* is the step-size parameter. Moreover, the choice of action *a_t_* in state *s_t_* follows exploitation and exploration policies, a widely used algorithm is the ϵ -greedy algorithm [34], which is defined as

(17) $$a\leftarrow \left\{ \begin{array}{l} \arg\underset{a}{\max}Q\left(s,a\right)\;\mathrm{with}\;\mathrm{probability}\;1-\epsilon \\ \mathrm{random}\;\mathrm{action}\;\mathrm{with}\;\mathrm{probability}\;\epsilon \end{array}\right.$$

Here, *ϵ* is the exploration rate. However, in larger networks, complex states and optional actions make Q-learning maintain a large Q-table and slow convergence speed, which limits the application scenario. Thus, we apply Q-learning techniques with a DNN parameterized by ***θ*** as the action-value function approximator to learn the optimal policies, thus called DQN [35]. To accelerate the learning convergence rate, we adopt double DQN [36] with three key techniques.

(1) Replay Memory: At each time step, BS observes the state **s***_t_*, determines resource allocation **a***_t_*, and broadcasts it to all V2V users. After the execution of the V2V links, the BS gets the corresponding reward *r_t_*_+1_, and the environment reaches the next state **s***_t_*_+1_. In this way, experience samples are formed, that is, (**s***_t_*, **a***_t_*, *r_t_*_+1_, **s***_t_*_+1_). Experience samples are stored in the replay memory. The replay memory accumulates the agent’s experiences over many episodes. After that, a mini-batch is uniformly sampled from memory for neural network training.

(2) Fixed Q-Target: As shown in Figure 2, our proposed scheme consists of double DQN, a target DQN and a training DQN, which have the same structure. The weight value ***θ***^target^ in the target network is updated by the training network weight ***θ***^train^ at regular intervals. This improves network stability and convergence.

(3) Action Selection–Evaluation Decoupling: In double DQN, the training DQN is used to select the optimal action for the next state, then the action selected by the training DQN is input and the next state into the target DQN to generate the target value to calculate the training loss. By changing the action of target DQN selection to training DQN selection, the risk of over-estimation of the Q value can be reduced.

After gathering sufficient experience samples, a mini-batch that consists of *D* experience samples is retrieved from the buffer to minimize the sum-squared error

(18) $$\sum_{t\in D}{\left[y_{t}-Q\left(s_{t},a_{t}|\theta^{\mathrm{train}}\right)\right]}^{2}$$

where *y_t_* is the target *Q*-value, accordingly, the *y_t_* can be respectively given by

(19) $$y_{t}=r_{t+1}+\gamma Q\left(s_{t+1},\underset{a_{t+1}}{\;\mathrm{argmax}}Q\left(s_{t+1},\;a_{t+1}|\theta^{\mathrm{train}}\right)|\theta^{\mathrm{target}}\right)$$

Then, the updating process for the BS double DQN can be written as [36]

(20) $$\theta^{\mathrm{train}}=\theta^{\mathrm{train}}+\beta \sum_{t\in D}\frac{\partial Q\left(s_{t},a_{t}|\theta^{\mathrm{train}}\right)}{\partial \theta^{\mathrm{train}}}\left[y_{t}-Q\left(s_{t},a_{t}|\theta^{\mathrm{train}}\right)\right]$$

where *β* is a nonnegative step size for each adjustment. Algorithm 1 summarizes the training process of the proposed scheme.
**Algorithm 1 Training Process for the Proposed Scheme**1: Input: double DQN structure, vehicular environment simulator and V2V pair delay requirements2: Output: double DQN networks’ weights3: Initialize: experience replay buffer, the weights of train DQN ***θ***^train^ and target DQN ***θ***^target^4: for each episode *j* = 1, 2,… do 5:   Start the V2X environment simulator for each episode 6:   Reset *L_n_*_,*t*_ = *L* and *T_n_*_,*t*_ = *T*_max_, for all n ∈ *N*7:  for each iteration step *t* = 1,2,… do 8:    Each V2V observes the observation **o***_n_*_,*t*_, sends it to BS 9:    BS based on the current state **s***_t_* = { **o**_1,*t*_,…,**o***_n_*_,*t*_,…}, select the action according to the      *ϵ* -greedy, then gets a reward *r_t_*_+1_, transforms to new state s*_t_*_+1_ 10:     Store transition (**s***_t_*, **a***_t_*, *r_t_*_+1_, **s***_t_*_+1_) into experience replay buffer 11:     Sample a mini-batch of *D* transition samples from experience replay buffer12:     Calculate the target value according to Equation (19) 13:     Update *θ*^train^ according to Equation (20)14:     Update ***θ***^target^ by setting ***θ***^target^ = ***θ***^train^ every *K* steps 15:  end for 16: end for

## 5. Simulation Results and Analysis

In this section, we provide the simulation results to demonstrate the performance of the proposed resource allocation method.

### 5.1. Simulation Settings

We considered a single-cell scenario. BS is located in the center of the region. The simulation setup we used was based on the urban case in 3GPP TR 36.885 [30]. We followed the main simulation setup in [22]. The main simulation parameters are summarized in Table 1, and the channel models of V2V and V2I links are shown in Table 2.

The double DQN for BS consists of three fully connected hidden layers, containing 1200, 800, and 600 neurons, respectively. The activation function used by the hidden layer of double DQN networks is ReLu *f*(*x*) = max(0, *x*). The RMSProp optimizer [37] is used to update network parameters. The discount factor of the double DQN algorithm is set to 0.05, and the learning rate is set to 0.001. Moreover, ***θ***^target^ is updated with ***θ***^train^ every 500 steps.

We trained the whole neural network for 4000 episodes and fixed the payload size for V2V links during the training phase to be 1060 bytes, which is varied during the test phase.

### 5.2. Performance Comparisons under Different Parameters

To verify the effectiveness of the algorithm in this paper, we compared the proposed algorithm with the following three algorithms.

(1) meta-DRL [22]: in this scheme, using DQN to solve the problem of spectrum resource allocation, and applying deep deterministic policy gradient (DDPG) to solve the problem of continuous power allocation.

(2) Brute-Force method: the action (including channel and power) is searched exhaustively to maximize the rate of V2V links.

(3) Random method: the channel and power are randomly selected.

Since we used the interference power measurement to design a partial CSI resource allocation mechanism, to prove the performance advantage of partial CSI, we compared it with complete CSI.

**Figure 3:**
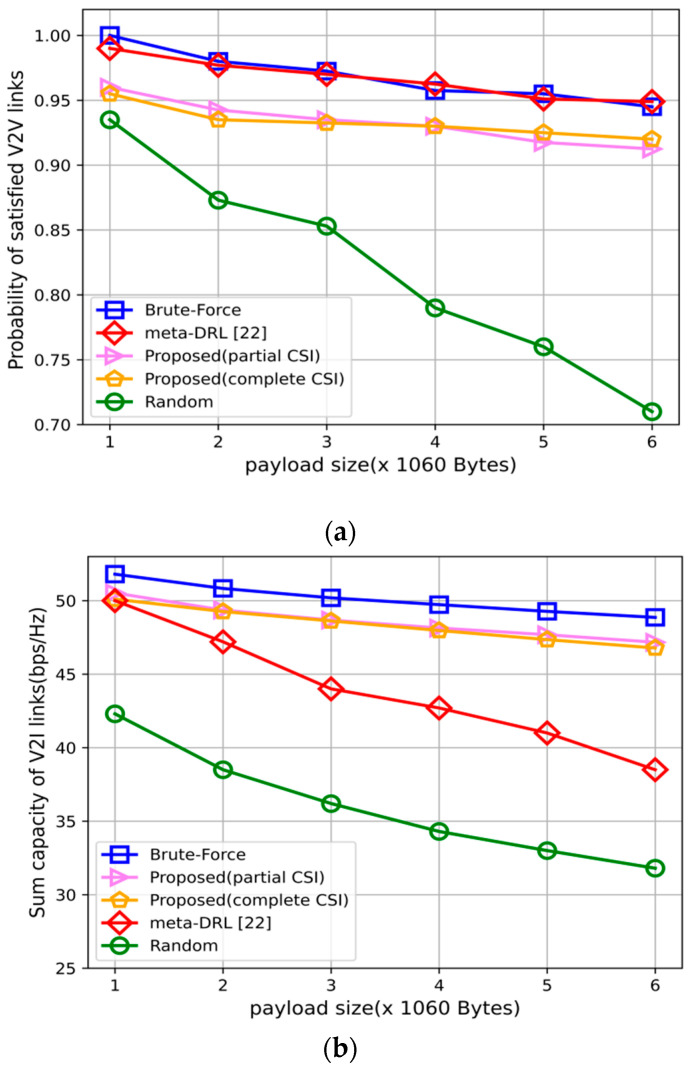
Performance comparisons with different payload sizes. (**a**) V2V payload transmission success probability with varying payload sizes. (**b**) Sum capacity performance of V2I links with varying payload sizes.

Figure 3 shows the changes in the V2V link’s successful transmission probability, V2I links throughput as the payload changes. Figure 3a reflects that as the payload size increases, the probability of successful transmission of the V2V links of all schemes decreases, including brute-force. This is because more information needs to be transmitted within the same delay constraint, so performance will decrease. However, it is found that the method in this paper is very close to brute-force and far superior to the random scheme. Since [22] considers the power control of the V2V links, the successful transmission probability of the V2V links is slightly higher than the scheme proposed in this paper. Furthermore, as can be seen from Figure 3a,b, the partial CSI proposed in this paper has comparable performance to complete CSI in meeting the V2V links delay.

Figure 3b reflects that as the payload increases, the total throughput of the V2I link gradually decreases. This is because when the V2V payload increases, in order to obtain higher rewards, the BS will select actions to increase the V2V rate to meet the delay constraint, which will increase the interference of the V2I links and cause its rate to decrease. However, it can be seen that the throughput of the V2I links obtained by the proposed scheme is still higher than that of the random scheme and has comparable performance to the complete CSI scheme. Furthermore, the proposed scheme outperforms the literature [22] in terms of V2I throughput. In summary, the scheme proposed in this paper is close to brute-force in terms of the V2V links’ successful transmission probability and the throughput of the V2I links, which is better than the random scheme and has comparable performance to complete CSI.

**Figure 4:**
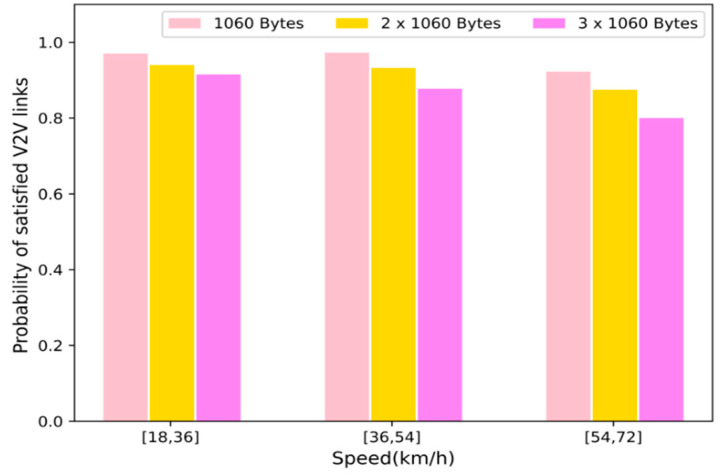
V2V payload transmission success probability with varying vehicle speeds.

Figure 4 shows the change in the probability of successful transmission of the V2V links as the vehicle speed increases. It can be seen from the figure that the probability of successful transmission of the V2V links in the proposed scheme decreases. This is because compared to the low-speed state, when the vehicle is in the high-speed state, the environmental state changes more significantly, leading to higher observation uncertainty and reducing the learning efficiency. Therefore, when the vehicle speed gradually increases, the V2V links’ successful transmission probability declines. However, the proposed scheme can still maintain a higher probability of successful transmission, which shows that the proposed algorithm has better stability in a highly dynamic environment.

**Figure 5:**
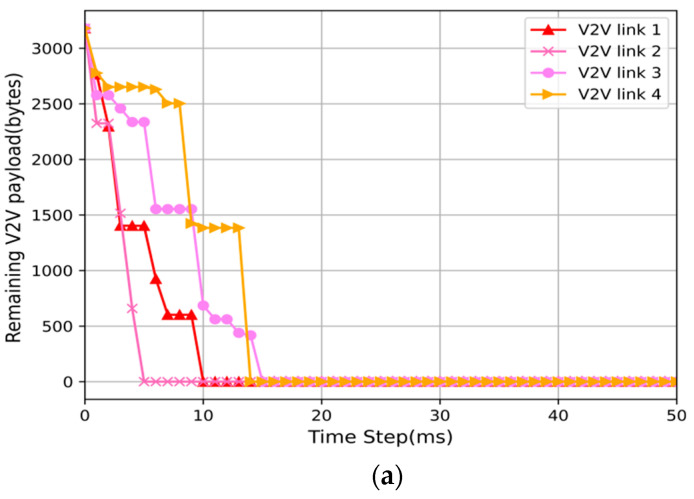

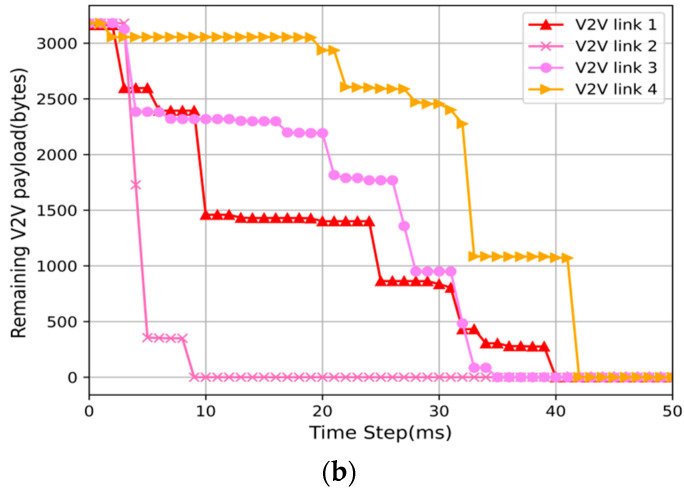
Remaining V2V payload comparisons between proposed and random schemes. (**a**) The remaining payload of the proposed. (**b**) The remaining payload of the random.

In order to more clearly explain the reason why the solution in this article is better than the random solution, we randomly selected an episode in the test and plotted the changes in the remaining load of the V2V links over time. Among them, the delay constraint T = 100 ms, and the payload size is 3 × 1060 bytes. Since in the randomly selected episode, all V2V links in the proposed scheme and the random scheme have completed the transmission task within 50ms, we only show the data within 0–50 ms. Comparing Figure 5a,b, it can be seen that although all V2V links of the proposed scheme and the random scheme have completed data transmission within the delay constraint, the transmission time required by the scheme of this paper is much shorter than that of the random scheme. As shown in Figure 5a, all vehicles in the scheme in this paper are transmitted within 15ms, while the random scheme in Figure 5b is completed within 42 ms. This shows that the solution in this paper is more suitable for transmitting delay sensitive services and meeting the delay requirements of the V2V links.

**Figure 6:**
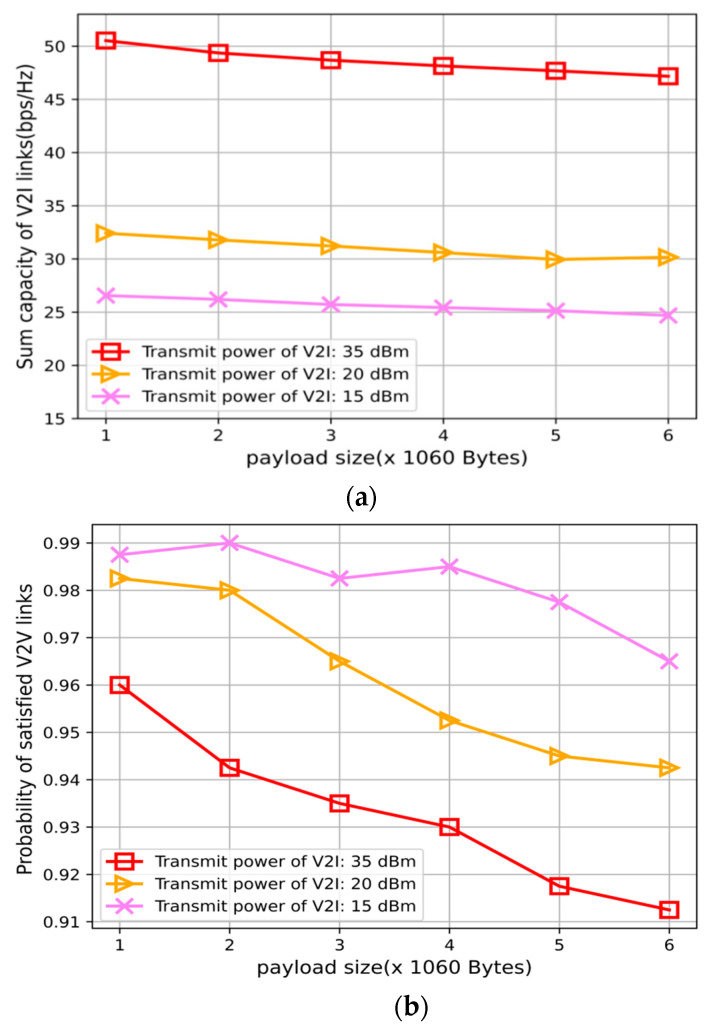
Performance comparisons with varying power. (**a**)Sum capacity performance of V2I links with varying power. (**b**) V2V payload transmission success probability with varying power.

Figure 6 reflects the impact of V2I power changes on V2I throughput and the probability of successful V2V link transmission. It can be seen that with the increase in V2I power, the throughput of the V2 link increases, and at the same time, the interference to the V2V links increases, so the probability of successful transmission of the V2V links decreases. Therefore, it is necessary to reasonably set the power of the V2I links to meet the throughput requirements of the V2I links and the delay requirements of the V2V links.

## 6. Conclusions

In this article, we developed a DRL-based resource sharing scheme in the underlay approach of mode 3, in which the V2V links reuse the V2I links spectrum. Our goal is to improve the throughput of V2I links while ensuring the V2V links delay constraint. In particular, to reduce the signaling overhead for the BS to acquire complete CSI in mode 3, an intelligent resource allocation strategy based on partial CSI is proposed. The BS only allocates a spectrum based on the feedback CSI, which significantly reduces the signaling overhead. The simulation results show that compared with other methods, the proposed scheme can meet the V2V links delay constraint and has a higher V2I links throughput, and the proposed partial CSI scheme has comparable performance as the complete CSI scheme.

## Figures and Tables

**Table 1:** Simulation parameters.

Parameters	Values
Carrier frequency	2 GHz
Subcarrier bandwidth	1 MHz
BS antenna height	25 m
BS antenna gain	8 dBi
BS receive noise figure	5 dB
Vehicle antenna height	1.5 m
Vehicle antenna gain	3 dBi
Vehicle receive noise figure	9 dB
Transmit power of V2I	35 dBm
Transmit power of V2V	23 dBm
Number of V2I links	4
Number of V2V pairs	4
[*λ*_c_, *λ_v_*]	[0.1, 0.9]
Noise power	−114 dBm
Vehicle speed	50 km/h
Latency constraint of V2V links	100 ms

**Table 2:** Channel models.

Parameters	V2I Link	V2V Link
Path loss model	128.1 + 37.6log_10_(*d*), *d* in km	WINNER + B1
Shadowing distribution	Log-normal	Log-normal
Shadowing standard deviation	8 dB	3 dB
Decorrelation distance	50 m	10 m
Fast fading	Rayleigh fading	Rayleigh fading

## Data Availability

Not applicable.
